# Editorial: Enzyme design methodologies to enhance enzymatic yield

**DOI:** 10.3389/fbioe.2023.1271980

**Published:** 2023-08-29

**Authors:** Ashish Runthala, Silambarasan Tamil Selvan, Thenkrishnan Kumaraguru

**Affiliations:** ^1^ Department of Biotechnology, Koneru Lakshmaiah Education Foundation, Vijayawada, India; ^2^ Department of Microbiology, School of Allied Health Sciences, VIMS Hospital Campus, Vinayaka Missions Research Foundation (DU), Salem, India; ^3^ Department of Organic Synthesis and Process Chemistry, CSIR-Indian Institute of Chemical Technology, Hyderabad, India; ^4^ Academy of Scientific and Innovative Research, Ghaziabad, India

**Keywords:** directed evolution, superenzyme, rational design approach, nature-inspired algorithm, extremophile, phi29 DNA polymerase, pectate lyase (PEL), loop replacement

The field of enzyme design methodologies has emerged as a promising avenue for enhancing enzymatic yield, enabling the optimization of enzyme performance for various industrial and scientific applications. These methodologies encompass a range of computational and experimental techniques aimed at tailoring enzyme properties, such as activity, selectivity, stability, and yield. By harnessing the power of rational design, directed evolution, and protein engineering, researchers can engineer enzymes with improved catalytic efficiency and substrate specificity, ultimately leading to more efficient and sustainable enzymatic processes. Drawing insights from protein structure and function, coupled with computational tools, rational design strives to craft enzymes with customized functions and improved effectiveness. This methodology facilitates purposeful adjustments to enzyme active sites, substrate binding regions, and overall structure, resulting in amplified catalytic activity, substrate specificity, stability, and other essential attributes. The strategic implementation of rational design principles not only expedites the creation of innovative biocatalysts but also drives progress across diverse sectors, from pharmaceuticals to sustainable energy, through the advancement of biotechnological solutions.

The generic protocol for creating a superenzyme involves screening for a more potent sequence variant. Subsequent steps include employing a series of intricate strategies such as selecting the most advantageous protein, identifying functionally significant and insignificant positions, mapping energetically and structurally allowed variations, tracing more favorable mutations, and experimentally validating the entire methodology. Although the recent machine-/deep-learning based strategies have reliably enhanced the accuracy of some of these sub-problems, this multifaceted approach is necessary to achieve the desired enhancement in enzyme function and performance. The enzyme design strategy heavily relies on iteratively tuned sets of computational algorithms and intensive experimental protocols, and evolving an enzyme sequence can be a challenging and intricate process, requiring the decoding of the enzyme’s sequence and structural details, particularly at the active site. Biology and industrial researchers have extensively explored the structure, function, and molecular network of proteins to identify key sites for customizing the involved promiscuous positions. Although this approach continues to be a significant subject in the realm of biological research, its widespread adoption faces obstacles in the form of academic, conceptual, and financial challenges. Concentrating on addressing some of these concerns, the primary focus of this Research Topic is to showcase the latest developments in the field, exemplifying the advanced methodologies and making them more accessible to researchers across various related disciplines. The articles included in this Research Topic encompass a variety of approaches, including protein engineering, directed evolution, rational design, and computational methods, with the aim of optimizing enzymatic processes.

This Research Topic comprises four articles, and mainly focuses on pioneering strategies and advancements in enzyme design, underscoring their pivotal role in propelling the advancement of biocatalysis and biotechnological applications. The first article, “*Rational design of enzyme activity and enantioselectivity*,” thoroughly reviews enzyme design methodologies that improve their biochemical properties, including stability in organic solvents, kinetic properties, temperature optimum, thermostability, and substrate specificity, in a controlled and predictable manner. To predict the potential mutants with the desired traits and mutate the key sites through site-directed mutagenesis, this approach majorly relies on decoding the detailed relationship between the structure and functional roles of an enzyme. As the enzyme structure-function relationship is a highly complex and dynamic system, designing functionally improved molecules is much more difficult than designing stable proteins. On basis of the detailed enzyme structure and its mechanism of a protein, this method scans the mutational landscape and results in the top-ranked mutations, likely to be more favorable. Song et al. and others present an overview of rational design methodologies used to engineer enzyme functionalities such as enantioselectivity and activity, including protein sequence alignment, steric hindrance and remodelling interaction networks, protein dynamics, computational design, and machine learning approaches, as well as their limitations and future prospects.

The second article, titled “*Nature-inspired Enzyme Engineering and Sustainable Catalysis: Biochemical Clues from the World of Plants and Extremophiles*,” provides a comprehensive overview of the underexplored potential of plant enzymes, particularly extremozymes, for industrial applications. Due to their sessile nature, plants encounter a diverse array of biotic and abiotic stresses, and resultantly, plants have evolved several stress-response mechanisms, such as producing enzymes to cope up with stress. Although microbial extremozymes have been extensively researched, algae and plants also have been recently shown to produce the extremozymes to survive against the natural/experimental stress, and it certainly holds a great promise for industrial applications. This review focuses on the examination of characteristic plant enzymes, such as carbonic anhydrase, ascorbate peroxidase, glycoside hydrolases, papain, among others, with a particular emphasis on their stress-tolerant properties and potential for further enhancement through enzyme engineering. Moreover, the review exemplifies intriguing instances of plant-derived enzymes, holding promising potential for industrial applications.

The third article titled “*Improved single-cell genome amplification by a high-efficiency phi29 DNA polymerase*” addresses the problem of uneven and incomplete genome amplification, and low amplification efficiency, of the Single-cell genome amplification (SGA). Superenzyme HotJa Phi29 DNA Polymerase is developed, and augmented with process engineering, it exhibits significantly improved coverage (99.75%) for commercial probiotic samples and at a bit higher temperature (40°C), and its sampling coverage reached 93.59%. The results demonstrate its potential for large-scale single-cell sequencing since it is 2.03 times more efficient, 10.89 times less expensive, and also more efficient than wild-type phi29 DNA polymerase.

The last article titled “*Improvement of optimum pH and specific activity of pectate lyase from Bacillus RN.1 using loop replacement*” exemplifies the application of the design strategies for the functional enhancement of the papermaking enzyme pectate lyase, but whose industrial usage is severely constrained by its poor alkaline resistance. In papermaking, pectate lyases are utilized to break down pectin, a plant cell wall component, thereby enhancing paper quality and characteristics. Song et al. and coworkers cloned the alkaline pectate lyase (BspPel) gene of *Bacillus* RN.1 into *Escherichia coli* BL21 (DE3) and replaced the 250–261 loop with the 268–279 loop of Pel4-N, additionally incorporating the mutation R260S to increase its activity and alkaline tolerance. Molecular dynamics simulation of the modelled wildtype and mutant protein at different temperatures indicates that the loop region with mutations is more flexible, enhancing the flexibility of the substrate-binding pocket and the overall yield. Showing remarkable stability for a wide range of pH from 3.0–11.0, the recombinant pectate lyase is found to be 4.4-fold more active at a pH of 11.0°C and 60°C. The study thus demonstrates an effective methodology for enhancing the alkaline tolerance of enzymes, offering the potential to expand the possible applications of different enzymes.

## Future perspectives

The field of enzyme design is in constant evolution, with researchers continuously developing innovative methodologies to meet the growing demands of industrial processes and sustainable solutions. Researchers endeavor to tailor the biochemical reactions, as well as its required structural perquisites, for enhancing the key enzyme properties, including efficiency, specificity, and stability, ultimately leading to higher yields in various biotechnological applications. Enzyme engineering approaches have been used for a variety of applications, including, the designing of novel enzymes/mutants for enhancing the cellular yield under constrained conditions through pathway/metabolic engineering, generating improved antibodies from scaffolds against the new constantly evolving disease variants, as well as therapeutic enzymes against various diseases, degrading/cleaning the environmentally hazardous molecules/polymers remediations, we still need significant advancements for the following key requirements:

1. Decoding the minimal set of key positions, mediating/aiding the enzyme function.
2. Faster and cheaper genetic diversification method for building sufficiently diverse mutant libraries.
3. Integration of robustly scoring computational methods to predict the most promising mutations and resultant mutant behavior and intelligently guide the process.
4. Robust assessment of the mutant library.
5. Quicker and more accurate experimental screening and selection methodology for tracing the most beneficial mutations.

The studies contribute to a broader understanding of enzymology and its practical applications, paving the way for more efficient and eco-friendly processes across various industries. As novel techniques, including Alphafold and CRISPR-Cas for precise gene editing, continue to advance, directed evolution is becoming a more potent, reliable and flexible approach to tailor biomolecules with precise characteristics. Despite these advancements, the enzyme design approaches are still not guaranteed to be effective due to unknown biological parameters guiding the function, cryptic sequence-functional relationship, complexity of the sequence space, and limited knowledge to trace its functionally crucial zone, although it has been becoming more and more promising. Hence, considering the high uncertainty and experimental cost, it is advisable to conduct focused, target-oriented research, adopt the right technology during the initial stages, and conduct a thorough market survey, and robust proofreading for enhancing the experimental accuracy.

